# Review of Pyorrhea Alveolaris Literature

**Published:** 1906-07-15

**Authors:** G. C. Bowles

**Affiliations:** Detroit, Mich.


### REVIEW OF PYORRHEA ALVEOLARIS LITERATURE.

BY G. C. BOWLES, D.D.S., OF DETROIT, MICH.

[Read before the Detroit Dental Society, March 8th, 190

To turn a telescope over a year's dental literature, then reverse the instrument, look into the large end and reduce the image to the compass of an evening's paper is a task to stagger one. Happily for this committee it contains a "Knight of the Pastepot and Shears," whose daily pleasure it is to thus compress tons of verbiage to ounces of thought. This wise provision obviates the necessity of acknowledging defeat at the outset. My assignment is "pyorrhea alveolaris."

"What is pyorrhea alveolaris? asks Dr. Eugene S. Talbot, in the November 1905 *Cosmos*, in an article entitled, "Interstitial Gingivitis." He answers it thus: "A second tooth erupts; interstitial gingivitis is set up to absorb the alveolar process, this is not pyorrhea alveolaris. Teeth are extracted interstitial gingivitis is set up to produce absorption and fill the cavity with new bone, this is not pyorrhea alveolaris. Wedging of teeth, gold bands extending under free margin of gums, the extra stress put upon roots carrying bridges, tartar, foreign deposits, local irritations, all set up interstitial gingivitis; and these inflammations may continue for a life time, or until the tooth has exfoliated, but if there be be no puss germs in the mouth, pyorrhea alveolaris cannot take place. Everyone has interstitial gingivitis, only about 10 percent have pyorrhea alveolaris. Interstitial gingivitis is a disease, pyorrhea is not a disease, but the result of a disease. The two pathologic conditions are separate and distinct. In the study of the two conditions the terms interstitial gingivitis and pyorrhea alveolaris should be used in their proper places. To which I would say Amen.

Interstitial gingivitis he divides into two classes, local, due to "anything and everything that will produce a local irritation," and constitutional, due to "faulty elimination." Gastro-intestinal fermentation is the great cause of interstitial gingivitis, due to faulty elimination and auto-intoxication."

In the January 1905 *Items of Interest*, Dr. D. D. Smith, of Philadelphia, in a paper entitled "Six years' work in Oral Prophylaxis," says: "Alveolar pyorrhea is not properly classed as a disease. Its very definition—'flow of pus'—implies that it should be taken from the category of diseases and placed among the inflammations. Properly defined, alveolar pyorrhea is an inflammation within the confines of the pericemental membrane and contiguous tissue; an inflammation due wholly to the irritation resulting from stagnant septic matter adherent to the surfaces, necks and roots of natural teeth. It need no longer be said that these are 'constitutional conditions,' complex in their manifestations."

The discussion following Dr. Smith's paper well epitomizes the chaotic state of the profession's mind on this subject of pyorrhea alveolaris, and makes one long for a Moses to lead us out of this wilderness of the opinionater into the promised land of the scientific investigator.

The magazines have contained many articles on pyorrhea alveolaris during the past year, but a brief glance at this discussion, will, in a convenient form, present practically all that has appeared on the subject, says Dr. S. Freeman, "Allow me to state that pyorrhea alveolaris is a disease, due to systemic conditions, with local manifestations, and can only be arrested when the proper medicaments are applied, or through treatment given to the patient to improve the general health, and treating locally the diseased condition of the teeth and gums."

Dr. Lenox Curtis, "The man who treats pyorrhea alveolaris as a disease will get left. The gums may be diseased, but the disease that causes it is syphilis and I know that for I have examined fifteen hundred persons that have had it. It is inherited more times than it is acquired."

Dr. Register, "I do not think pyorrhea is a disease but merely a phase of a disease. We have only one membrane between the cementum and the alveolus, which supplies both, but is especially put there for the benefit of the cementum. The disease will be found to be an affection first of the pericementum. When the pericementum is gone, it is gone for all time. The vulnerable point of infection is at the gingival border. There exists a recess for the reception of infinite small quantities of granulated calcic matter and food debris and general matter, all septic laden. This is the initial cause of gingivitis that may run into the pericementum through its different stages, terminating in pyorrhea alveolaris."

Dr. Schamberg, "Some of the speakers have told us it is of purely local origin, and there are those who maintain that it is caused in the main by constitutional difficulties. Any man who hopes to find one single cause for pyorrhea alveolaris is working in the wrong direction, just as he would be if he tried to cure all cases of pyorrhea alveolaris by any one method. It has been said that the savage is free from pyorrhea alveolaris. Does he use a prophylactic tooth brush or any other tooth brush? No, but he leads a different kind of life from ours, and that explains it all."

So much for the published views as to the nature and cause of pyorrhea alveolaris. All admit that irritating substances on or about the roots of the teeth are at least one of the manifestations of pyorrhea alveolaris. On this phase of the subject Dr. Edward C. Kirk has something to say in the July 1905 *Cosmos*, in an article entitled "Tartar upon the Roots of Teeth, its Formation and Removal." He says, There are two distinct varieties or classes of calcareous concretions upon the teeth. Frist, those upon the exposed portions of the tooth-crowns, salivary in origin. Second, a variety of deposit differing in characteristics and found upon the roots of teeth, called sanguinary tartar or serumal tartar from the belief that it was derived directly from the blood or blood plasma.'' Some writers have questioned the origin of the second class of tartar from the blood. In reference to this, Dr. Kirk rays. "So far as my own observations are concerned, I have satisfied myself beyond all reasonable doubt that calcareous deposits do form upon the roots of teeth in instances where the saliva is absolutely excluded as a possible factor. I have extracted a number of teeth in which careful exploration showed no break whatever in the gingival attachment, and yet a calcareous deposit was found upon the root. In such cases I have, however, noted a distinct difference in the character of the deposits as compared with those cases in which the attachment of the gum and pericementum was destroyed, as in the case of pyorrheal pocket. In the former instance the deposit was at the apex or near it, was more nodular and less firmly adherent than in the latter, and is always associated with positive evidence of existing or previous suppurative inflammation about the root. Tartar deposits contain calcium and magnesium phosphates and carbonates, also the ammonia-magnesium phosphate known as 'tripple phosphate' in combination with organic matter. The great solubility of phosphate of lime in even the weakest acids is very extraordinary. Phosphate of lime mechanically suspended in water is speedily dissolved by passing a copious stream of carbonic acid gas through it. The carbonic acid in the blood plasma is an important factor in holding the earthy phosphates in solution. The extremely weak nature of the chemical affinity between carbonic acid and the earthy phosphates permits of its ready decomposition from a variety of causes. Puss is more highly alkaline than the blood plasma. Either the increased alkalinity of puss formation, or the presence of ammonia as an end product of putrefaction would result in the precipitation of earthy phosphates from the blood plasma, poured into an area where such chemical changes were taking place. Tartar formation includes the chemical and physical modification which the precipitated earthy salts undergo by combination with a colloidal element. This latter element is furnished by the mucin of the saliva. The albuminous constituent of the blood plasma, or the protoplasmic matters entering as a constituent of puss. It is the organic colloidal compound of tartar and especially of the serumal variety, which gives density and adhesiveness to the masses with which we have to deal in the treatment of pyorrheal cases."

In a communication entitled, "Uratic deposits upon the Roots of Teeth," Dr. Julio Endleman, (*Cosmos*, 1905,) throws a further light upon this subject of tartar formation. Says he: "Diseases of mal-nutrition may be divided into two groups, those due to insufficient oxidation, the hyper-acid diathesis, and those due to excessive oxidation, the hypo-acid diathesis. The former is characterized by the presence within the fluids of the body of waste products, acid in character, due principally to an excess of nitrogenous principles in the organisms. This excess of nitrogen is converted, eventually, into uric acid, which combines with the bases sodium and potassium. The resulting urates circulate freely, perhaps, through every organ and tissue of the body. They are maintained in solution by the alkalinity of the liquor sanguinis, and are precipitated in the presence of an acid, or under conditions of marked decrease in alkalinity. Therefore, in the gouty diathesis, uratic deposits are the immediate result of the formation of acid substances, or of decreased alkalinity about the parts which become the seat of such deposits. During rest and inactivity the reaction of the tissues is alkaline. During prolonged activity the reaction changes into one of marked activity, due to the breaking down of the proteid elements, producing lactic acid among other compounds. Samples of tartar analized by Professors Congdon and Brubaker, for Professor Pierce, were found to consist chiefly of sodium urate, free uric acid and calcium phosphate. Sodium urate is precipitated in an acid medium, calcium phosphate in an alkaline medium. What is the rational of their deposit upon the roots of a tooth? A tooth through mal-occlusion or other cause is subjected to undue stress. This leads to excessive oxidation, decrease of alkalinity and probably to the formation of lactic acid through the breaking down of the complex proteid molecules of the pericemental membrane and finally to the precipitation upon that area of urate salts. This deposit sets up an irritation which leads to cell death, infection and purulent inflammation, with the concomitant breaking down of the tissues. Puss, with extravasated serum and disorganized proteid elements has a degree of alkalinity greater than that of the blood, and therefore causes a precipitation of the phosphates upon the nucleus of uratic salts.

This last paragraph Endleman quotes from Kirk, to whom he is largely indebted for his views, and from which it will be seen that both agree with Dr. Talbot that pyorrhea alveolaris is due to both, local and systemic causes.

In these days, when it is the custom to attribute all the ills to which the teeth are heir, to the artificial conditions of modern civilization, it is interesting, if not absolutely encouraging, to find facts showing that the same conditions exist among communities, little, if at all, affected by the so-called artificiallity of civilized life, and that they have existed among civilized and uncivilized people at least as far back as evidence goes. It is a mistake to regard the primitive life and habits of the savage, as natural, and the life and habits of our modern time as artificial and unnatural. The one enchanging law of nature is change; change from the simple to the complex, from the complex back to the simple, in ever-recurring cycles. This is true of the atom, of man, of the solar and the stellar systems. Everything is in a state of evolution or devolution. Man is evolving. From the primordial cell upward every step in advance might have been called artificial as compared to the former state, and as a matter of fact, throughout historic time, we know that it has been so-called. The Golden Age has always been in the past the life of a people existing in caves, making no provision for the morrow, fighting single-handed all the forces of Nature which are arrayed against them, is natural as infancy is natural; so is our present civilization, which may be likened to adult life. Its con-comitant science is conscientiously bringing us into closer harmony with Nature, it studies her laws, and is widening our friendly relations with them, setting them over against our natural enemies, drudgery, cold, famine, disease, etc. To one who thus believes that the Golden Age is approaching and not receding, it is a real pleasure to come across an article demonstrating that pyorrhea alveolaris, for instance, is not as many proclaim, a product of our modern life, and that it is not necessarily due to meat eating or disuse of the teeth.

Dr. W. J. Younger, than whom, perhaps, no one is more competent to write on the subject, in an article in the October 1905 *Dental Review*, entitled "Pyorrhea Alveolaris in the Times of the Pharaohs and the present Egyptians," has such an article. He says: "It has been my theory that the exciting cause of pyorrhea alveolaris is the lodgment of hard particles of food and other insoluble substances between the free margin of the gum and the crevices of the teeth, such as form husks of cereals, covering of beans, peas, lentels, fragments from fibrous plants, as cellery, asparagus seeds of berries, earthy particles, sands, etc., which, during the act of mastication and the formation of the bolus, were forced by the tongue into the spaces where they remain trapped until the irritation and inflammation set up by their presence extended to the alveolar edge, and so prepared the way for bacterial invasion and resultant pyorrheal infection." D. E. T. Darby, of Philadelphia, has been quoted as saying that the Egyptians were singularly immune from decay and pyorrhea, due to the fact that the people lived chiefly on raw wheat, grinding up the whole grain with their teeth. Dr. Younger, who had made two visits to Egypt, says: "Feeling sure that I had detected the swollen and irregular gum margins indicative of pyorrheal alveolaris. I was astonished at this declaration. I determined, consequently, upon my next visit to Egypt to make critical examinations of the mouths of living Egyptians as well as of the mummies." The opportunity presented. In two clinics at Cairo he examined ninety cases of both sexes, and of ages from twelve to sixty two, and with the exception of four, all under twenty-three years of age, everyone had pyorrhea in more or less advanced condition. These people were practically vegetarians. A man of sixty-two had never tasted animal food; none ate it more than once a week, the great majority but once a month, some only once in six months.

Thinking that unsanitary surroundings might predispose to this remarkable prevalence of pyorrhea, he went to Fayoum, four hours by rail from Cairo. "We proceeded to several distant villages and spent the greater part of the day in making examinations among the Fallaheen, that is the agriculturists. Their diet is entirely vegetable, except on Fridays and certain Feastdays. Their teeth showed abundent evidence of wear by mastication and in many of the older inhabitants their crowns were worn almost entirely away. Yet in all the mouths examined, young men and old, there was not *one* found in which the ravages of pyorrhea were not plainly visible."

Speaking of his studies among the mummies made under the supervision and with the assistance of Dr. G. Elliott Smith, keeper of the museum at Cairo, he says: "In none of these subjects, some dating 7,000 years before B. C., did we find any trace of dental art, whilst the necessity for it, as evidenced by badly decayed teeth, the openings in the alveolar wall, caused by alveolar abscess and the erosive effects of pyorrhea were abundant."

"The conclusions, then, to be drawn from all these examinations are, First, that pyorrhea alveolaris, as well as caries, existed in the time of the Pharaohs, and is rampant among the Egyptians of the present day. Second, that the science and art of treating pathological conditions of the teeth was unknown to the Egyptians, certainly to the time of Cleopatra and the Roman occupation; and that all stories to the contrary are but fictions of the imagination, fairy tales of impossible story tellers."

Dr. Losada, of Spain, speaking before the American Dental Club of Paris (*Cosmos*, February 1905), says: "People in my country—poor people, who eat hard bread and vegetables only, and very little meat, have pyorrhea in teeth ground down by exercise. In the Royal College of Surgeons, London, I examined a number of prehistoric crania. They had pyorrhea, for there was tartar on some of their teeth." In the *Cosmos* for January 1905, note is made of a report of pyorrhea alveolaris in India by Major Andrew Buchanan. "The writer has observed a considerable number of cases of pyorrhea alveolaris in India. In a population of about one thousand there were two hundred cases."

This is an interesting subject, too large to be properly handled in the time allowed. However, I think the quotations pretty well show the drift of present thought on the subject. Regarding treatment, there is much more unanimity of opinion. Briefly stated, it may be said to be, thorough removal of all deposits, the steadying of all loose teeth by splints, bands, ligatures or other means, followed by what is coming to be generally known as prophylaxis treatment. Concerning the removal of deposits, Dr. Younger says: "That must be done thoroughly—not as well as you can—you must do it thoroughly, because if a particle of deposit remains you will not get perfect tissue adhesion. The scraping of the roots in itself will produce a denudition of the alveolus, with concomitant growth of granulations. The use of lactic acid aids the removal of the disorganized tissue that may remain, and helps to denude the surfaces not reached by the instruments; also helps to stop the openings of the canaliculæ. There is no better germicide."

Dr. Good has devised a long, curved, blunt-pointed needle for the hypodermic syringe with which to introduce the lactic acid. He protects the gums with cotton rolls, inserts the needle to the bottom of the pocket and floods it thoroughly. Dr. Kirk tells us why lactic acid is preferable for this use. "It has a decided advantage of directly dissolving the calcarous salts without chemically decomposing them and forming insoluble compounds, whereas sulphuric acid, decomposes the calcium phosphate, forming calcium sulphate or Plaster of Paris. which is practically insoluble. Tri-chloracetic acid is valuable, but its escharotic property tends to harden the organic matrix of the tartar. "Dr. Kirk has devised a dentate scaler. It acts by breaking up the deposit into small particles, disintegrating it so that its removal is accomplished with much less force and greater certainty than a continuous edge instrument."

Loose teeth should be supported and put into a condition of physiological rest. "The conditions to be avoided are all those which would tend to the formation of areas of relative or actual acidity around the pericementum." If as stated, uratic salts are the first deposited, if they are only deposited in an acid medium, and if this acid medium is induced by undue stress or over stimulation of the peri-dental membrane, then the necessity for supporting loose teeth is obvious.

In conclusion, it appears to the writer that pyorrhea alveolaris is no longer regarded with the hopelessness of former times. Some writers, Dr. Talbot for one, still hold that constitutional treatment is demanded in some of its forms, but there appears to be a growing conviction that with all irritating and infectious substances removed from about the teeth, and the maintenance of a vigorous oral asepsis, pyorrhea alveolaris, if not too far advanced, is curable.

